# Pathologic Response and Survival after Neoadjuvant Chemotherapy with Bevacizumab Followed by Surgery for Clinical Stage II/IIIA Nonsquamous Non-Small-Cell Lung Cancer: Results from a Phase II Feasibility Study (NAVAL)

**DOI:** 10.3390/cancers16132363

**Published:** 2024-06-27

**Authors:** Yasuhiro Tsutani, Yoshihiro Miyata, Kenji Suzuki, Fumihiro Tanaka, Hiroyuki Ito, Yoshinori Yamashita, Morihito Okada

**Affiliations:** 1 Department of Surgical Oncology, Hiroshima University, Hiroshima 734-8551, Japan; ymiyata@hiroshima-u.ac.jp (Y.M.); morihito@hiroshima-u.ac.jp (M.O.); 2 Division of Thoracic Surgery, Department of Surgery, Kindai University Faculty of Medicine, Osaka-Sayama 589-8511, Japan; 3 Department of Thoracic Surgery, Juntendo University School of Medicine, Tokyo 113-8421, Japan; kjsuzuki@juntendo.ac.jp; 4 Second Department of Surgery (Chest Surgery), University of Occupational and Environmental Health, Kitakyushu 807-8555, Japan; ftanaka@med.uoeh-u.ac.jp; 5 Department of Thoracic Surgery, Kanagawa Cancer Center, Yokohama 241-8515, Japan; h-ito@kcch.jp; 6 Department of Thoracic Surgery, Kure Medical Center/Chugoku Cancer Center, Kure 737-0023, Japan; yamashita.yoshinori.tr@mail.hosp.go.jp

**Keywords:** non-small-cell lung cancer, neoadjuvant chemotherapy, bevacizumab, pathologic response, survival

## Abstract

**Simple Summary:**

This study of nonsquamous lung cancer patients undergoing neoadjuvant chemotherapy with bevacizumab followed by surgery revealed that 20% were pathologic responders, experiencing 100% 5-year survival rates. In contrast, the 80% nonresponders demonstrated significantly lower rates. Pathologic response emerged as a survival predictor, indicating prolonged post-surgery survival for responders, while nonresponders required additional therapy for improved outcomes.

**Abstract:**

The objective of this study was to evaluate the relationship between pathologic response and survival in patients with clinical stage II/IIIA nonsquamous non-small-cell lung cancer (NSCLC) who intended to undergo neoadjuvant chemotherapy with bevacizumab, followed by surgery. In this phase II NAVAL study evaluating the feasibility of neoadjuvant chemotherapy with cisplatin (75 mg/m^2^), pemetrexed (500 mg/m^2^), and bevacizumab (15 mg/kg), followed by surgery, progression-free survival (PFS) and overall survival (OS) were assessed as the secondary endpoints. Patients were categorized based on the proportion of residual viable primary tumor in the resected specimen after neoadjuvant chemotherapy: those with residual tumor in less than one-third were classified as pathologic responders, the rest as nonresponders. Of the 30 patients, 25 underwent surgical resection after three cycles of neoadjuvant chemotherapy with bevacizumab; 5 did not undergo surgery. Among all 30 patients, the rates of 2- and 5-year PFS were 41.5% and 34.6%, respectively, and the rates of 2- and 5-year OS were 70.0% and 60.0%, respectively. A total of 6 patients (20%) were classified as pathologic responders; the other 24 (80%), as nonresponders. The five-year PFS differed significantly between pathologic responders (100%) and nonresponders (17.5%; *p* = 0.002). The five-year OS also differed significantly between pathologic responders (100%) and nonresponders (43.5%; *p* = 0.006). Pathologic response seems to be a predictor of survival. Long-term survival after surgery is expected for pathologic responders, whereas additional therapy is needed for nonresponders.

## 1. Introduction

The surgical outcomes of stage II/IIIA resectable non-small-cell lung cancer (NSCLC) are unsatisfactory; the rates of 5-year disease-free survival (DFS) and 5-year overall survival (OS) are 49.7% and 64.1%, respectively, for stage IIA disease; 46.3% and 56.1%, respectively, for stage IIB disease; and 27.8% and 47.9%, respectively, for stage IIIA disease [1]. Adjuvant chemotherapy is recommended as standard treatment for patients with resected stage II/III NSCLC who have not received neoadjuvant chemotherapy [2]. On the other hand, neoadjuvant chemotherapy theoretically could provide additional benefits for patients with NSCLC, such as reducing the local tumor and metastatic lymph node cells, eliminating metastases from small tumors, and demonstrating the sensitivity of the tumor to the chemotherapy regimen [3,4].

We hypothesized that neoadjuvant chemotherapy with bevacizumab might improve survival in patients with stage II/IIIA NSCLC because the combination of carboplatin, paclitaxel, and bevacizumab improved survival more than carboplatin and paclitaxel alone in patients with recurrent or advanced NSCLC [5]. Therefore, we conducted a phase II study (NAVAL) of the safety and feasibility of neoadjuvant cisplatin, pemetrexed, and bevacizumab, followed by surgery, in patients with clinical stage II/IIIA nonsquamous NSCLC [6].

Major pathologic response (MPR), which is defined as 10% or less residual viable tumor after neoadjuvant chemotherapy, has been proposed as a predictor of survival [7,8]. However, data on the pathologic response and survival after neoadjuvant chemotherapy with bevacizumab are sparse [9]. Therefore, in this phase II NAVAL feasibility study, we also investigated the relationship between pathologic response and survival in patients with clinical stage II/IIIA nonsquamous NSCLC who intended to undergo neoadjuvant treatment with cisplatin, pemetrexed, and bevacizumab, followed by surgery.

## 2. Materials and Methods

### 2.1. Patients

The inclusion and exclusion criteria of the NAVAL study were previously described [7]. Key inclusion criteria were as follows: resectable clinical stage II/IIIA nonsquamous NSCLC, according to the Tumor Node Metastasis (TNM) Classification of Malignant Tumors, 7th edition [10]; patient age of 20–75 years; Eastern Cooperative Oncology Group (ECOG) performance status score of 0–1; no previous chemotherapy, radiotherapy, or surgery for lung cancer; predicted postoperative forced expiratory volume of >1000 mL/s; and adequate hematologic, hepatic, and renal function. Examples of key exclusion criteria were uncontrollable systemic disease, a history of active double cancer, and a history of pregnancy or lactation. We also tried to exclude patients who expected to undergo pneumonectomy.

The protocol was approved by the institutional review board and the ethics committee at the participating institution (28 May 2010 RIN-183, Hiroshima University Hospital), and all patients provided written informed consent to the procedures. Data were collected, managed, and analyzed by the Advanced Clinical Trial Chest Surgery Group in Japan. This study is registered with the University Hospital Medical Information Network, number 000004278.

### 2.2. Study Treatment

Patients received standard supplementation with oral folic acid (0.5 mg daily) and intramuscular vitamin B12 (1 mg every 9 weeks) at least 7 days before the first dose of pemetrexed. Three cycles of cisplatin (75 mg/m^2^), pemetrexed (500 mg/m^2^), and bevacizumab (15 mg/kg) were administered in 21-day intervals.

After completing the three cycles of neoadjuvant chemotherapy, the patients were assessed for their response, and the disease was restaged. If disease control was achieved, we planned for surgery 6–9 weeks after the last dose of bevacizumab. To be eligible for surgery, patients had to have recovered from any severe adverse effects; have an ECOG performance status of 0–1; have a predicted postoperative forced expiratory volume of >1000 mL/s; and have adequate hematologic, hepatic, and renal function. In cases of hematologic toxicity, surgery could be delayed up to 9 weeks until the absolute neutrophil count was 2 × 10^9^/L, the platelet count was 10 × 10^9^/L, the hemoglobin level was 9 g/dL, the creatinine level was 1.2 mg/dL, and the ratio of aspartate aminotransferase to alanine aminotransferase was 100 IU/L.

Curative lung resection, which included en bloc removal of the affected lobe, was performed as lobectomy or bilobectomy (but not sublobar resection); these procedures included segmentectomy or removal of additional lung parenchyma with adjacent structures, if necessary, with complete hilar and mediastinal lymph node dissections. Moreover, the bronchial stump was covered with viable tissue, such as an intercostal muscle flap.

Adjuvant chemotherapy or radiation therapy after complete resection was not performed in this study.

### 2.3. Assessment of Pathologic Response

We categorized the pathologic response based on the 7th edition of the General Rules of Clinical and Pathological Records of Lung Cancer in Japan as follows [11]:

- No pathologic response (Ef 0): no pathologic changes in the cancer cells in the resected specimen.
- Slight pathologic response (Ef 1): viable cancer cells remaining in more than one-third of the resected specimen.
- Moderate pathologic response (Ef 2): viable cancer cells remaining in up to one-third of the resected specimen.
- Complete pathologic response (Ef 3): no viable cancer cells in the resected specimen.

Pathologic responses were confirmed by pathologists from each participating institution. Patients who had a response of Ef 2 or Ef 3 after neoadjuvant chemotherapy were classified as pathologic responders; the others, including patients who did not undergo protocol surgery, were classified as nonresponders.

### 2.4. Follow-up Evaluation

All patients were monitored starting from the day of registration. Postoperative follow-up for the first 2 years included (1) physical examinations and chest radiography every 3 months and (2) chest and abdominal computed tomography (CT) scans every 6 months. Subsequently, patients underwent physical examinations and chest radiography every 6 months, supplemented by annual CT scans.

### 2.5. Statistical Analysis

Unless otherwise stated, we calculated the data as either a number and percentage or a median and range. We used the chi-square test to compare frequencies of categorical variables and Fisher’s exact test for small samples. To compare continuous variables, we applied *t* tests and the Mann–Whitney *U* test. We defined progression-free survival (PFS) as the time from the day of registration to either the first event (disease progression in patients who did not undergo surgery, postoperative recurrence, or death from any cause) or the last follow-up visit. We defined OS as the time from the day of registration to either death from any cause or the last follow-up visit. To analyze the durations of both PFS and OS, we applied the Kaplan–Meier method, and to assess differences, we used the log-rank test. A *p* value of <0.05 indicated statistical significance. To analyze the data, we used JMP 15.0 (SAS Institute, Cary, NC, USA).

## 3. Results

**Figure 1:**
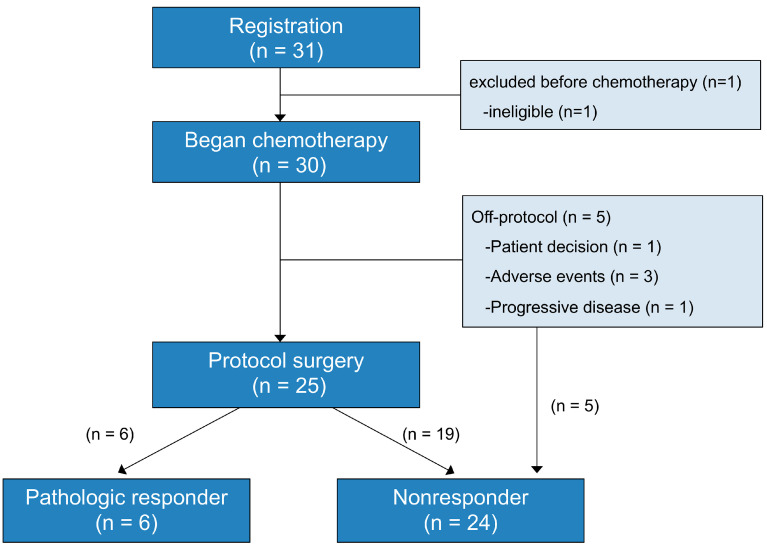
Schema of the study.

The study schema is shown in Figure 1. Of the 30 patients in the study, 25 (83.3%) underwent the protocol surgical resection after neoadjuvant treatment; 5 (16.7%) did not undergo surgery.

The patients’ characteristics are listed in Table 1. The median length of the follow-up was 61.1 months. Clinical stage IIIA was diagnosed in 22 patients (73.3%), and 8 (26.7%) harbored epidermal growth factor receptor (EGFR) mutations.

Of the 30 patients, 6 (20%) achieved a moderate (Ef 2) or complete (Ef 3) pathologic response and were classified as pathologic responders. The other 24 (80%) patients—19 who underwent surgery and 5 who did not—were classified as nonresponders. Table 2 lists the pathologic findings of the 25 patients who underwent the protocol surgery.

The characteristics of the pathologic responders were compared with those of the nonresponders (Table 3). EGFR mutation status differed significantly between the pathologic responders and nonresponders (*p* = 0.039): none of the pathologic responders harbored EGFR mutations, whereas eight (33.3%) of the nonresponders did.

**Figure 2:**
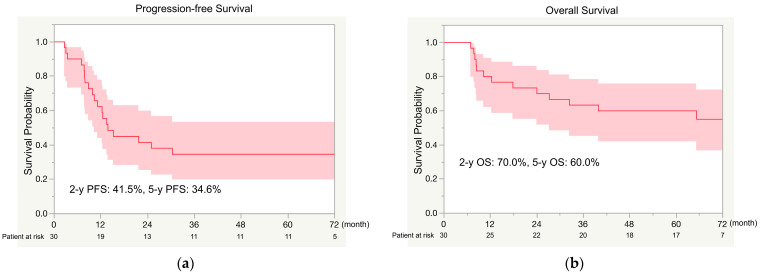
Progression-free survival (PFS) (**a**) and overall survival (OS) (**b**) among all study patients. 2-y, 2-year; 5-y, 5-year.

**Figure 3:**
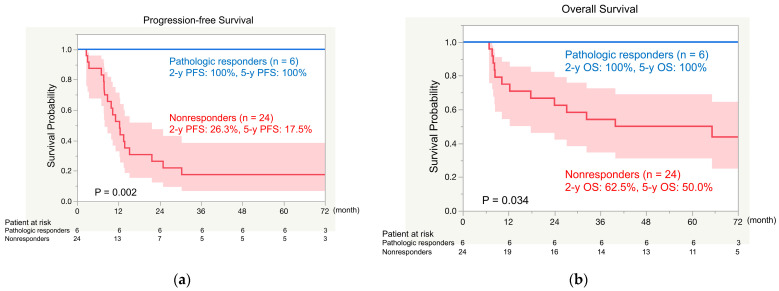
Comparison of progression-free survival (PFS) (**a**) and overall survival (OS) (**b**) between pathologic responders and nonresponders after neoadjuvant treatment with cisplatin, pemetrexed, and bevacizumab. 2-y, 2-year; 5-y, 5-year.

Among the 30 patients, the rates of 2- and 5-year PFS were 41.5% and 31.6%, respectively (Figure 2a), and the rates of 2- and 5-year OS were 70.0% and 60.0%, respectively (Figure 2b). The rates of 5-year PFS differed significantly between the pathologic responders (100%) and nonresponders (17.5%; *p* = 0.002; Figure 3a). Rates of 5-year OS also differed significantly between pathologic responders (100%) and nonresponders (50.0%; *p* = 0.034; Figure 3b).

## 4. Discussion

Miyata et al. previously reported that the neoadjuvant regimen of cisplatin, pemetrexed, and bevacizumab was safe and feasible, with an 83% rate of complete resection in the phase II NAVAL study [7]. This study concerned the long-term outcomes after neoadjuvant cisplatin, pemetrexed, and bevacizumab, with or without surgery, for clinical stage II/IIIA nonsquamous NSCLC in patients who participated in the NAVAL study. Although the 5-year PFS among all 30 patients was only 31.6%, the 5-year OS of 60.0% was relatively favorable in comparison with the historical data in Japan [2].

We focused on the relationship between pathologic response and survival in this study. Although the MPR has been proposed as a predictor of survival [8], the use of the pathologic response instead of OS as a primary endpoint is still controversial in phase III clinical trials of neoadjuvant therapy. We demonstrated that 25 of 30 enrolled patients achieved complete resection after three cycles of neoadjuvant chemotherapy with bevacizumab and that 6 (20%) of the 30 patients were pathologic responders, but 24 (80%) were nonresponders. Both the PFS and OS differed significantly between the pathologic responders and nonresponders, and none of the responders developed postoperative recurrence. These results strongly suggest that the pathologic response is a predictor of survival in patients who receive neoadjuvant cisplatin, pemetrexed, and bevacizumab for clinical stage II/III NSCLC.

We defined the pathologic responders as patients for whom less than one-third of the resected specimen had residual viable primary tumor after neoadjuvant chemotherapy on the basis of Japanese guidelines [11]. This cutoff differed from that of the MPR. In a phase II study of neoadjuvant cisplatin, docetaxel, and bevacizumab, 11 (22%) of 50 patients had MPRs, and the investigators found significant differences in the rates of 3-year recurrence-free survival (91% vs. 48%; *p* = 0.024) and 3-year OS (100% vs. 49%; *p* = 0.011) between patients who had MPR and those who underwent surgical resection but did not have MPR, respectively [9]. In several phase III trials of neoadjuvant treatment, the primary endpoint was the MPR or complete pathologic response [12,13,14], but the optimal cutoff value of residual viable tumor should be evaluated further.

In this study, the EGFR mutation status differed significantly between the responders and nonresponders; the nonresponders harbored EGFR mutations. The combination of cisplatin, pemetrexed, and bevacizumab was previously reported to be effective in patients with advanced or recurrent nonsquamous NSCLC [15,16,17]; in terms of pathologic response after neoadjuvant therapy, however, the effect may be limited in patients with nonsquamous NSCLC who have EGFR mutations. The Japan Intergroup Trial of Pemetrexed Adjuvant Chemotherapy for Completely Resected Nonsquamous Non-Small-Cell Lung Cancer (JIPANG) study was a phase III trial in which pemetrexed plus cisplatin was compared with vinorelbine plus cisplatin as adjuvant chemotherapy for completed resected stage II/IIIA nonsquamous NSCLC; in the subgroup analysis of patients harboring EGFR mutations, recurrence-free survival and OS tended to be better in patients who received vinorelbine plus cisplatin than among those who received pemetrexed plus cisplatin [18,19]. These findings suggest that survival might be improved by a strategy different from cisplatin and pemetrexed, with or without bevacizumab, as perioperative therapy for NSCLC with EGFR mutations.

Recently, adjuvant osimertinib with or without chemotherapy greatly improved DFS, with a hazard ratio of 0.17 and OS with a hazard ratio of 0.49, in patients with stage II/IIIA NSCLC with EGFR mutations [20,21,22,23]. Also, neoadjuvant osimertinib with or without chemotherapy is being compared with chemotherapy for resectable stage II/IIIB NSCLC with EGFR mutations [12]. EGFR-tyrosine kinase inhibitors for resectable NSCLC with EGFR mutations seem to improve survival. In addition, several phase III studies of neoadjuvant or adjuvant immunotherapy for resectable NSCLC mainly without driver gene mutations are ongoing [13,14,15,24,25,26,27,28,29,30]. Nonresponders who do not harbor driver gene mutations after neoadjuvant cisplatin, pemetrexed, and bevacizumab may benefit from adjuvant immunotherapy.

One limitation of this study is the relatively small patient cohort. Nonetheless, we were able to clearly demonstrate the relationship between the pathologic response and survival in the patients with stage II/IIIA nonsquamous NSCLC after neoadjuvant chemotherapy with bevacizumab. Another limitation is the use of our specific pathological assessment method rather than the MPR-based assessment that has gained international acceptance in recent years. However, since our definition of pathologic responders includes patients with 33% or fewer viable cancer cells, the outcomes for patients with MPR are encompassed within the responder category. Specifically, the 5-year PFS and 5-year OS rates for the patients with MPR were both 100%, indicating a favorable association between MPR and survival outcomes.

## 5. Conclusions

The pathologic response after neoadjuvant cisplatin, pemetrexed, and bevacizumab for resectable stage II/IIIA nonsquamous NSCLC seems to be a predictor of survival. Long-term survival after surgery is expected for pathologic responders, whereas additional therapy is needed for nonresponders.

## Figures and Tables

**Table 1:** Patients’ characteristics.

Variable		*n* = 30
Age, median (range)		64 (54–71)
Male sex		17 (56.7%)
Smoking history		21 (70.0%)
Clinical stage	IIA	5 (16.7%)
	IIB	3 (10.0%)
	IIIA	22 (73.3%)
Histologic diagnosis of adenocarcinoma (before treatment)		30 (100%)
EGFR mutation status	Ex19 del	3 (10.0%)
	Ex21 L858R	5 (16.7%)
	Wild type	20 (67%)
	Unknown	2 (6.7%)
Race	Japanese	30 (100%)

EGFR, epidermal growth factor receptor; Ex, exon; del, deletion; L, leucine; R, arginine.

**Table 2:** Surgical procedures and pathologic findings.

Variable		*n* = 25
Procedure	Lobectomy	22 (88.8%)
	Bilobectomy	3 (12.0%)
Histologic diagnosis	Adenocarcinoma	23 (92.0%)
	LCNEC	1 (4.0%)
	NSCLC	1 (4.0%)
Clinical stage	0	2 (8.0%)
	IA	2 (8.0%)
	IB	4 (16.0%)
	IIA	1 (4.0%)
	IIB	0 (0%)
	IIIA	13 (52.0%)
	IIIB	0 (0%)
	IV	1 (4.0%)
Pathologic response *	Ef 0	1 (4.0%)
	Ef 1a	13 (52.0%)
	Ef 1b	5 (20.0%)
	Ef 2	3 (12.0%)
	Ef 3	3 (12.0%)

LCNEC, large-cell neuroendocrine carcinoma; NSCLC, non-small-cell lung cancer. * According to the 7th edition of the General Rules of Clinical and Pathological Records of Lung Cancer [11].

**Table 3:** Characteristics of pathologic responders and nonresponders.

Variable		Pathologic Responders (*n* = 6)	Nonresponders * (*n* = 24)	*p* Value
Median age, years		64 (range, 61–71)	63 (range, 54–71)	0.262
Male sex		4 (66.7%)	13 (54.2%)	0.577
Smoking history		5 (83.3%)	16 (66.7%)	0.405
Clinical stage				0.269
	IIA	0 (0%)	5 (20.8%)	
	IIB	1 (16.7%)	2 (8.3%)	
	IIIA	5 (83.3%)	17 (70.8%)	
EGFR mutation status	Positive	0 (0%)	8 (33.3%)	0.039
	Wild type/unknown	6 (100%)	16 (66.7%)	
Radiologic response				0.281
	Partial response	4 (66.7%)	7 (29.2%)	
	Stable disease	2 (33.3%)	13 (54.2%)	
	Progressive disease	0 (0%)	3 (12.5%)	
	Not evaluable	0 (0%)	1 (4.2%)	

EGFR, epidermal growth factor receptor. * Nonresponders included 19 patients with poor response and 5 patients who did not undergo surgery.

## Data Availability

The data that support the findings of this study are available from the corresponding author upon reasonable request.
